# Family-Based Association Study of Pulmonary Function in a Population in Northeast Asia

**DOI:** 10.1371/journal.pone.0139716

**Published:** 2015-10-02

**Authors:** Ho-Young Son, Seong-Wook Sohn, Sun-Hwa Im, Hyun-Jin Kim, Mi Kyeong Lee, Bayasgalan Gombojav, Hyouk-Soo Kwon, Daniel S. Park, Hyung-Lae Kim, Kyung-Up Min, Joohon Sung, Jeong-Sun Seo, Jong-Il Kim

**Affiliations:** 1 Department of Biochemistry and Molecular Biology, Seoul National University College of Medicine, Seoul, Republic of Korea; 2 Department of Internal Medicine, Dongguk University Ilsan Hospital, Goyang, Republic of Korea; 3 Department of Obstetrics and Gynecology, College of Medicine, Chung-Ang University, Seoul, Republic of Korea; 4 Genomic Medicine Institute, Medical Research Center, Seoul National University, Seoul, Republic of Korea; 5 Department of Epidemiology and Institute of Environment and Health, School of Public Health, Seoul National University, Seoul, Republic of Korea; 6 Department of Internal Medicine, Asan Medical Center, Seoul, Republic of Korea; 7 Department of Molecular and Cellular Biology, Harvard University, Cambridge, Massachusetts, United States of America; 8 Department of Biochemistry, Ewha Womans University, School of Medicine, Seoul, Republic of Korea; 9 Department of Internal Medicine, Seoul National University College of Medicine, Seoul, Republic of Korea; 10 Department of Biomedical Sciences, Seoul National University Graduate School, Seoul, Republic of Korea; 11 Cancer Research Institute, Seoul National University College of Medicine, Seoul, Republic of Korea; Yale School of Public Health, UNITED STATES

## Abstract

The spirometric measurement of pulmonary function by measuring the forced expiratory volume in one second (FEV_1_) is a heritable trait that reflects the physiological condition of the lung and airways. Genome-wide linkage and association studies have identified a number of genes and genetic loci associated with pulmonary function. However, limited numbers of studies have been reported for Asian populations. In this study, we aimed to investigate genetic evidence of pulmonary function in a population in northeast Asia. We conducted a family-based association test with 706 GENDISCAN study participants from 72 Mongolian families to determine candidate genetic determinants of pulmonary function. For the replication, we chose seven candidate single nucleotide polymorphisms (SNPs) from the 5 loci, and tested 1062 SNPs for association with FEV_1_ from 2,729 subjects of the Korea Healthy Twin study. We identified *TMEM132C* as a potential candidate gene at 12q24.3, which is a previously reported locus of asthma and spirometric indices. We also found two adjacent candidate genes (*UNC93A* and *TTLL2*) in the 6q27 region, which has been previously identified as a pulmonary function locus in the Framingham cohort study. Our findings suggest that novel candidate genes (*TMEM132C*, *UNC93A* and *TTLL2*) in two different regions are associated with pulmonary function in a population in northeast Asia.

## Introduction

Pulmonary function, which is commonly measured by spirometry, is a good index of the physiological condition of the lung and airways [1]. Forced expiratory volume in one second (FEV_1_) and its ratio to the forced vital capacity (FEV_1_/FVC) are used to predict population morbidity and mortality [2–4] and are used in the clinical diagnosis of chronic obstructive pulmonary disease (COPD) and asthma [5]. Family studies have reported COPD aggregation and significant heritability of spirometry-measured pulmonary function [1, 6, 7].

To discover genetic loci related with pulmonary function and COPD, several linkage studies have been conducted using the quantitative spirometry measures FEV_1_, FVC, and the FEV_1_/FVC ratio [8, 9]. The Framingham Heart cohort study suggested that the linkage locus for FEV_1_ was on the terminus region of chromosome 6q [10, 11].

In recent years, genome-wide association studies (GWAS) have revealed various genes showing significant associations with pulmonary function [12, 13]. Moreover, large-scale GWAS meta-analyses from CHARGE and the SpiroMeta consortium each had more than 20,000 participants and identified novel genome-wide significant loci associated with pulmonary function in the general population [14–16]. Although numerous genome-wide linkage and association studies have revealed a number of genes and genetic loci associated with pulmonary function, these genetic results explain only a small proportion of the genetic variation needed to estimate the heritability and variance of the trait [17]. In addition, most previous association studies on pulmonary function have focused on populations of European ancestry and not on those of Asian ancestry.

As part of the GENDISCAN (GENe DIScovery for Complex traits in isolated large families of Asian of Northeast) project, which was designed to investigate genetic loci associated with complex traits in extended rural families [18–20], we conducted a family based association study of pulmonary function in a Mongolian population. Subsequently, we validated the association between the candidate loci identified by our study and the pulmonary function phenotype in a population of Korean subjects to confirm the genetic evidence in two different populations.

## Materials and Methods

### Study design and population

From April to June 2006, a total of 2,008 participants for pulmonary function measurement were recruited in Dashbalbar, Dornod Province, Mongolia, as part of the GENDISCAN project [18, 20, 21], which was designed to identify genetic loci of complex traits in Asian populations. For this study, we selected 706 subjects from 72 large and extended pedigrees with complete genotyping. We extracted genomic DNA from peripheral blood leukocytes of all subjects according to standard protocols. Written informed consent was obtained from all participants, and our study protocols were approved by the institutional review board of Seoul National University (Approval number, H-0307-105-002). This study abided by the principles of the Declaration of Helsinki.

### Phenotype measurement

Spirometry was performed with a portable spirometer (MicroPlus spirometer, Micro Medical Ltd, Rochester, Kent, England) according to American Thoracic Society criteria [22]. FEV_1_ and FVC were measured, and FEV_1_ was used to evaluate airway obstruction. After several practice measurements with a trained technician, measurements for data collection were taken three times. Of these measurements, the greatest FEV_1_ values from acceptable tests for each subject were selected. The subjects were instructed to take complete inspirations and expirations that lasted approximately 3 s [23]. The predicted FEV_1_ and FVC values were obtained from the methods of Morris [24]. Smoking history data (current smoking, former smoking, and never smoked) was collected by questionnaire and the number of pack-year was calculated using the smoking amount (pack/day) and the duration (smoking years). Information on if the participants have any of the following respiratory diseases was also collected by questionnaire: chronic obstructive lung disease, chronic bronchitis, pneumonia, asthma or tuberculosis.

### Genome-wide SNP genotyping

Details of the genotyping methods used have been previously reported [18, 25]. In brief, 706 samples from 72 extended families were genotyped using an Illumina Human 610-Quad BeadChip kit (San Diego, CA). To maintain the quality of the genotyping data, we checked for genotyping error at several steps before analysis. SNPs with a call rate < 99%, an error rate > 1%, minor allele frequency < 1% and a Hardy-Weinberg equilibrium *P* < 1 × 10^−6^ were excluded.

### Statistics

Before the association test, the subject’s measurements of pulmonary function were adjusted for age, age^2^, sex, height, dummy variables of smoking status (current, former, never) and pack-year as covariates using the sequential oligogenic linkage analysis routines (SOLAR) package [26]. To reduce deviations from normality and the effect of outliers, we normalized the phenotypes using the inverse normal transformation option in the SOLAR package. We conducted genome-wide family-based association analysis using the additive model to investigate genetic regions that might influence pulmonary function using FBAT software version 2.0.3 [27].

### Replication test

We compared the results of the GENDISCAN study with data from the Korea Healthy Twin Study. The Healthy Twin Study is an ongoing cohort study of twins and their families that was initiated in 2005. The details of the study’s protocols, measurements, genotyping and imputation methods have been reported previously [28, 29, 30]. In brief, a total of 2,729 participants were recruited and genotyped using the Affymetrix Genome-wide Human SNP Array version 6.0 (Affymetrix Inc., Santa Clara, California, USA). The untyped SNPs were imputed to the HapMap3 phase 2 (JPT+CHB) and Korean HapMap (http://www.khapmap.org) reference panel using Beagle (University of Washington, Seattle, WA, USA). We selected 1062 SNPs for replication analysis that were located within 200 kb of the associated SNPs of discovery stage.

## Results

The descriptive characteristics of our discovery and replication subjects are presented in Table 1. In the discovery stage, 706 individuals from the 72 families from the Mongolian population were assessed. The majority (88.7%) ethnicity of this cohort was Buryats. In the replication study, 623 families from 2,729 Korean participants were included. The mean age of the Mongolian population was 30.3 years, and the mean age of Korean population was 44.2 years. The mean (S.D.) FEV_1_ values were 2.684 (0.717) and 2.915 (0.709), respectively. With respect to smoking status, Koreans were more likely to be current smokers (27.6%) and had higher mean pack-year (17.63) than the Mongolians.

**Table 1:** Descriptive characteristics of the participants.

	Discovery stage	Replication study
Subject information		
No. of families.	72	623
Total subjects number	706	2729
Age, years	30.3 ± 14.9	44.2 ± 13.1
Female, N (%)	366 (51.8)	1667 (61.1)
Height (cm)	156.2 ± 10.9	161.6 ± 8.5
FEV_1_ (L)	2.684 ± 0.717	2.915 ± 0.709
Smoking Status		
Never smokers, N (%)	565 (80)	1776 (65.1)
Current smokers, N (%)	129 (18.3)	754 (27.6)
Former smokers, N (%)	12 (1.7)	199 (7.3)
Smoking, Pack-years	8.77 ± 9.73	17.63 ± 16.99

Data represent the mean values ± standard deviation unless otherwise indicated.

FEV_1_, forced expiratory volume in one second.

**Fig 1:**
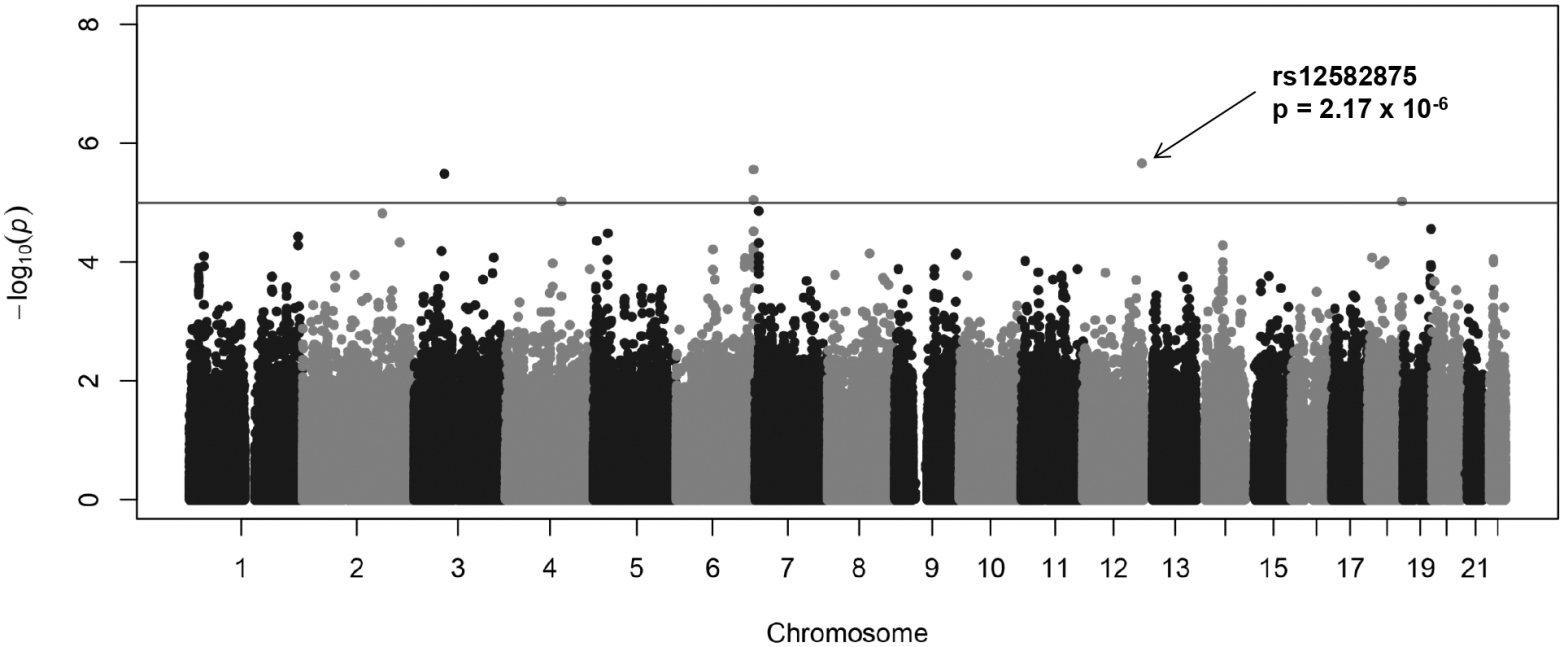
Manhattan plot of the genome-wide association signal with FEV_1_. The x-axis represents the SNP markers on each chromosome. y-axis shows the −log10(*P* value). The blue horizontal line represents the genome-wide suggestive threshold *P* = 1.0 × 10^−5^. The greatest *P* value (*P* = 2.17 × 10^−6^) was observed in rs12582875 on chromosome 12q24.3.

**Fig 2:**
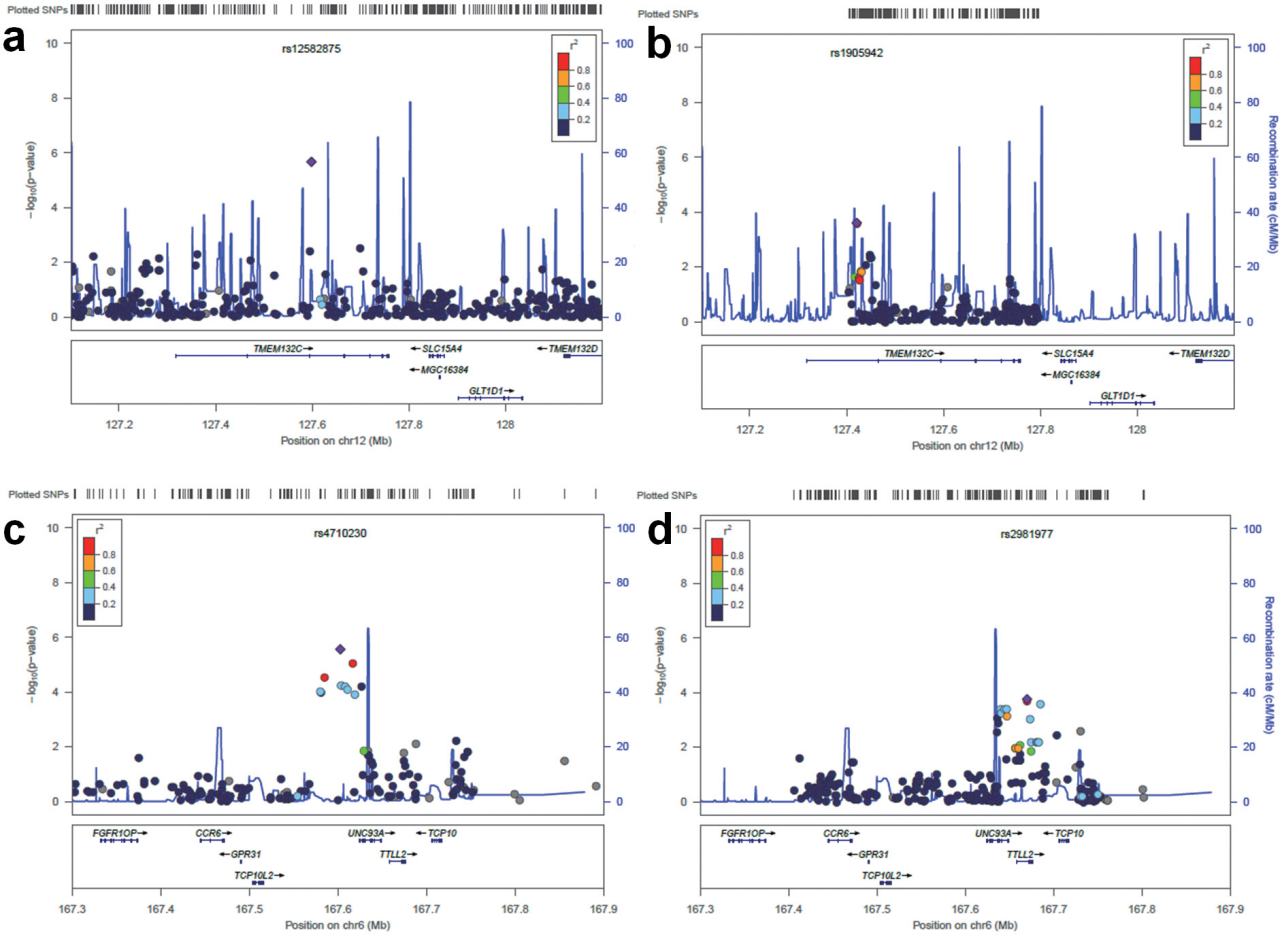
Regional plots for discovery and replication loci associated with FEV_1_. The purple diamonds indicate the most significant SNP of each region, and nearby SNPs are color coded according to the level of LD with the top SNP. The x-axis shows chromosomal position. The left y axis shows the significance of the association, and the right y-axis shows a recombination rate across the region. Estimated recombination rates from the 1000 Genome (JPT+CHB, hg18) database are plotted with the blue line to reflect the local LD structure. Discovery (**a**) and replication (**b**) result of the *TMEM132C* region on 12q24.3. Discovery (**c**) and replication (**d**) result of the *UNC93A* and *TTLL2* region on 6q27. The regional plots were created using LocusZoom.

The results of the family-based association between the genome-wide SNPs and FEV1 are shown in a Manhattan plot (Fig 1). Each of the approximately 510,000 SNPs is represented by single dot. Five regions were identified that contained seven suggestively associated SNPs with *P* values less than 1 × 10^−5^ (Table 2). The most significant association was observed in the intronic region of *TMEM132C* (rs12582875, *P* = 2.17 × 10^−6^) at 12q24.3 (Fig 2a). On chromosome 6q27, two SNPs (rs4710230 and rs3010558, *P* = 2.77 × 10^−6^ and *P* = 8.99 × 10^−6^, respectively) were located 21.7 Kb and 7.8 Kb upstream of *UNC93A* (Fig 2c). On chromosome 3p14.1, rs264676 (*P* = 3.28 × 10^−6^) was located in the *MAGI1* intron. We found one SNP (rs7504607) at 18q23, was associated with FEV_1_ phenotype, but has no candidate gene within 200 kb. At chromosome 4q27, rs6855113 and rs6831851 (both *P* = 9.48 × 10^−6^) were located 17kb and 33 kb upstream of the *MAD2L1* gene, respectively.

**Table 2:** Results of the family based association test for FEV_1_ (*P* value < 1.0 × 10^−5^) in the discovery stage.

Chr	SNP	Position*	Nearest gene	Nearest gene distance	Minor Allele	MAF	*P* value
12	rs12582875	127598647	*TMEM132C*	0	A	0.161	2.17 × 10^−6^
6	rs4710230	167603059	*UNC93A*	21733	T	0.387	2.77 × 10^−6^
6	rs3010558	167616938	*UNC93A*	7854	T	0.383	8.99 × 10^−6^
3	rs264676	65861112	*MAGI1*	0	G	0.307	3.28 × 10^−6^
18	rs7504607	73349291	*GALR1*	238207	C	0.458	9.45 × 10^−6^
4	rs6855113	121225082	*MAD2L1*	17621	T	0.079	9.48 × 10^−6^
4	rs6831851	121241185	*MAD2L1*	33724	C	0.079	9.48 × 10^−6^

* SNP positions are based on NCBI Build 36.

Chr, Chromosome; MAF, Minor allele frequency.

Because it is important to take into account the effect of the respiratory disease on pulmonary function, we verified if any participants had respiratory diseases. Thirty-two participants from the discovery stage had one or more conditions among 5 respiratory-related diseases (chronic obstructive lung disease, chronic bronchitis, pneumonia, asthma and tuberculosis). To find out the extent of these participants’ effect on the result, we excluded these participants with respiratory diseases and repeated the test for genetic association with FEV_1_. The result of the seven associated SNPs is shown in S1 Table.

For the replication study, we conducted an association test between the seven SNPs from the discovery stage and lung function in 2,729 subjects from 623 families from the Korea Healthy Twin study. For the different genotyping platforms, overlapping SNPs were not sufficient. Therefore, we performed imputation, in which 5 SNPs of the 7 associated SNPs from the discovery stage were included. However, we could not find any significant association (*P* value < 0.05) between these SNPs and lung function in the replication study. Of the five SNPs, we found that the allele frequency of four SNPs between the discovery and replication stage was significantly different (S2 Table). These different allele frequencies suggest that the genetic architecture of two populations might be quite different. Therefore, we considered a locus specific replication.

We selected a total of 1062 SNPs from the regions surrounding the seven candidate SNPs, including 200 kb up and downstream of the SNPs. The candidate regions with the most associated SNPs that have a *P* value < 1 × 10^−3^ are listed in Table 3. Twelve SNPs on and near three genes were shown to have associations with FEV_1_. Of the 225 SNPs near *TMEM132C*, rs1905942 and rs1112925 (*P* = 2.51 x 10^−4^) showed the highest association (Fig 2b). There was no LD between rs1112925 and the top SNPs of the discovery stage (rs12582875) because the two SNPs were located 177 kb away from each other. On chromosome 6, ten candidate SNPs were located within two adjacent genes (*UNC93A* and *TTLL2*) (Fig 2d). The six SNPs were located in the intronic region of *UNC93A*. rs2981977, rs3010556 and rs877653 were located in the *TTLL2* intron and exon, whereas rs3010562 is 9 kb downstream of this gene. These SNPs near *UNC93* and *TTLL2* were in LD, but there was no LD between these replicated SNPs and the most significant SNP (rs4710230) of the discovery stage.

**Table 3:** Replication result of the associated SNPs (*P* value < 1.0 × 10^−3^) in the Korea Healthy Twin Study.

Chr	SNP	Position*	Gene	Gene Position	Minor Allele	MAF	*P* value
6	rs2981977	167670140	*TTLL2*	Intron	G	0.307	1.76 × 10^−4^
6	rs3010556	167670167	*TTLL2*	Intron	G	0.308	2.10 × 10^−4^
12	rs1905942	127420950	*TMEM132C*	Intron	C	0.5	2.51 × 10^−4^
12	rs1112925	127421487	*TMEM132C*	Intron	A	0.5	2.51 × 10^−4^
6	rs3010562	167685241	*TTLL2*	Intergenic	C	0.477	2.64 × 10^−4^
6	rs4709162	167640315	*UNC93A*	Intron	A	0.473	4.03 × 10^−4^
6	rs4709165	167644640	*UNC93A*	Intron	G	0.459	4.09 × 10^−4^
6	rs4709167	167646922	*UNC93A*	Intron	C	0.459	4.09 × 10^−4^
6	rs3817767	167640832	*UNC93A*	Intron	C	0.464	5.74 × 10^−4^
6	rs2294236	167647304	*UNC93A*	Intron	A	0.278	7.17 × 10^−4^
6	rs3010550	167636618	*UNC93A*	Intron	C	0.329	8.82 × 10^−4^
6	rs877653	167673681	*TTLL2*	Exon	C	0.458	9.57 × 10^−4^

* SNP positions are based on NCBI Build 36.

Chr, Chromosome; MAF, Minor allele frequency.

## Discussion

We conducted a genome-wide association study to find genetic evidence of spirometric measures of pulmonary function in large extended families in a Mongolian population. In addition, we validated these candidate loci in 2,729 Korean individuals, and we could identify three candidate genes: *UNC93A*, *TTLL2* and *TMEC132C*.

*UNC93A* and *TTLL2* are located on chromosome 6q27, which is a region that has previously been reported to be associated with pulmonary function in many different studies. The linkage study in the Framingham cohorts revealed the greatest linkage peak at the q terminus of chromosome 6 for FEV_1_ [10]. Additional markers, significant evidence of linkage and family-based association were reported at 184.5 cM of chromosome 6 in 1,115 individuals from the 182 large extended Framingham Heart Study families [11]. This linkage result was replicated again in similar regions of chromosome 6 from a different study that used 100K SNP GeneChip [13]. Subsequently, secreted modular calcium-binding protein 2 (*SMOC2*) at 6q27 was selected as a candidate gene to study, and an association with the pulmonary function phenotype was reported [31]. However, little is known about the detailed molecular and physiological functions of *SMOC2*.

Consistent with these previous findings, we identified a suggestive association with FEV1 and the 6q27 region. The SNP with the greatest association (rs4710230, *P* = 2.77 × 10^−6^) on chromosome 6q27 was located 21.7 Kb upstream of *UNC93A*. Moreover, *TTLL2*, *TCP10L2*, *TCP10*, *GPR31* and *CCR6* were within 200 kb. Through the replication study, ten SNPs on or near *UNC93A* and *TTLL2* were validated. To find the functional relevance of the associated SNPs, we verified regulatory chromatin states using HaploReg v3. The region of rs4710230 was reported as a strong enhancer of *UNC93A* in HepG2 cells. The LD block of the replicated SNPs (rs4709265, rs4709167 and rs3817767) was reported as a strong and weak enhancer region. The rs2981977 and rs3010556 regions were reported as weak enhancers of *TTLL2* in HepG2 cells. A genome-wide association of methylation study reported that rs3010556 is significantly associated with the CpG (cg13033054) site in a *trans* manner [32]. rs877653 is located on third exon of *TTLL2* as a synonymous variant.

*UNC93A* and *UNC93B1* are human homologues to the *Caenorhabditis elegans (C*. *elegans) UNC93* gene. In *C*. *elegans*, *unc–93* is one of five genes in an interacting set (*unc–93*, *sup–9*, *sup–10*, *sup–11* and *sup–18*) involved in the contraction and coordination of muscle. Mutations in these genes produce the characteristic "rubber-band" phenotype in worms [33]. Little is known about the molecular function of human *UNC93A*. Human *UNC93B1*, however, has been identified to be related to left ventricular diastolic function, heart failure morbidity and mortality [34]. FEV_1_ is determined by airway obstruction and reflects the degree of airway obstruction in obstructive lung diseases, such as asthma and COPD. Asthma is a chronic inflammatory airway disease characterized pathologically by airway smooth muscle hypertrophy and contractility dysfunction. Because of the association between *UNC93* and muscle contraction, we posited that *UNC93* gene function might influence smooth muscle function in the airway. Moreover, murine *UNC93B* has been identified as a novel component of the innate and the adaptive immune response [35]. Asthma has also been regarded as an inflammatory disease mediated by immunologic T helper type 2 (Th2) lymphocytes and *UNC93* is thought to affect this immunologic balance. Therefore, SNPs in *UNC93* could be affecting pulmonary function by negatively influencing smooth muscle contraction and/or immunologic responses in COPD and asthma. The significant SNPs identified by the discovery and replication studies were located on different LD blocks. Each SNP in *UNC93A* might also have a separate effect on the FEV1 phenotype, so this gene by itself might be influencing pulmonary function in the general population.

Another candidate gene, *TTLL2* (Tubulin tyrosine ligase-like family, member 2), is thought to be related to tubulin glutamylase, which forms polyglutamate side chains on tubulin in airway epithelial cilia [36]. Previous studies have shown that *TTLL1* depletion resulted in a loss of tubulin glutamylation and disrupted the beating of airway cilia. Moreover *TTLL1* deficiency resulted in chronic sinusitis and abnormal development of spermatid flagella in mice [37]. From these previous results we postulated that *TTLL2* might be able to affect ciliary movement in the lung. The consequences of impaired ciliary function are abnormal mucus clearance from the airways and increased respiratory infection consistent with a condition such as COPD or asthma. *TTLL2* is located right next to the *UNC93A* gene, and the two genes are in the same LD block. Therefore, functional studies will be required to identify whether one or both of these genes influence pulmonary function.

In addition, we discovered *TMEM132C* as a novel candidate gene for FEV1 and pulmonary function. On chromosome 12, the SNP in the *TMEM132C* gene showed the highest association (rs12582875, p = 2.17 × 10^−6^) with FEV1 phenotype in the Mongolian population. We checked the regulatory chromatin states using HaploReg v3. However, no functional relevance was found in the region of the *TMEM132C* SNPs.

The molecular function of *TMEM132C* has not yet been identified. *TMEM132C* is a subfamily of the *TMEM132* gene family, which has 5 subtypes (*TMEM132A—E*). *TMEM132B* is located on chromosome 12q24.31, *TMEM132C* and *TMEM132D* are tandemly located on chromosome 12q24.32 – 12q24.33. Chromosome 12 terminus region (12q24.3) is a suggestive candidate locus for asthma and spirometric indices. Studies in various populations have reported an association between 12q24.3 regions and asthma [38–40]. Recently, rs2030436 in the *TMEM132D* gene was reported to have an association with lung function decline in a mild COPD genome-wide study [41]. Suggesting the relationship between pulmonary function and the *TMEM132* family, we concluded that the *TMEM132C* gene would be a novel candidate gene for the pulmonary function.

Three other candidate regions from the discovery stage were tested for replication. However, we could not find any association in the Korean population, and we suspect this may be a result of ethnic differences between the two populations.

There were several limitations of this study. First, we performed SNP specific replication test using imputed genotype data, but discovery associated SNPs were not replicated in the Korean population. These SNPs showed a different allele frequency between the discovery and replication populations, which suggests the possibility of different genetic architecture. For this reason, we conducted a locus specific replication test. Second, FVC was not evaluated in our study. Because we allowed the expiratory maneuver to last at least 3 seconds for a more accurate FEV1 measurement, full FVC efforts lasting at least 6 seconds could not be examined [23]. Third, the sample size of the discovery stage is rather small compared to other genome-wide association studies. Therefore, we used genome-wide suggestive *P* value instead of a Bonferroni adjustment.

However, several unique values in our study design might enable us to detect candidate results. First, we used large extended families in a rural isolated population, which has several advantages in genetic studies. An isolated population is suitable for genetic research because it can minimize the environmental component of the phenotype and has a restricted genetic heterogeneity [42]. Furthermore, extended multi-generation families with a small number of founders are known to increase the genetic power [43]. Second, the replication of the results in two different ethnic groups in Asia may provide solid evidence of association with respect to pulmonary function. As described above, most of the genetic studies of pulmonary function have been focused on Caucasian populations but not on Asian populations. The only exception was very recent report on the association of the 6p21 region with pulmonary function in Korea [30]. The difference between their results and our results might be due to the different study design (general population vs. large extended family) and ethnic group (Korean vs. Mongolian) in the discovery phase. Although Mongolia and Korea are located in northeast Asia, the geographical residences of these two populations are separated by approximately 1,700 km. Moreover, Mongolian lifestyle and dietary patterns are based on a nomadic culture, and Koreans have had an agricultural lifestyle for thousands of years.

In conclusion, our study focused on discovering a genetic determinant of pulmonary function in two northeast Asian populations. We found novel candidate genes (*UNC93A*, *TTLL2* and *TMEM132C*) in two regions that had been reported to show linkage and/or association with pulmonary function in Caucasians. Our results can be used for further functional studies of pulmonary function and provide more insight into genetic factors of pulmonary function in the general population.

## Supporting Information

S1 Table Family-based association result for FEV_1_ in the discovery stage (participants without known respiratory disease only). (DOCX)

S2 Table The comparison of the minor allele frequency of 7 associated SNPs in the discovery and replication study. (DOCX)
