# Supplementary material for: Family-Based Association Study of Pulmonary Function in a Population in Northeast Asia
Source: PLoS One. 2015 Oct 2;10(10):e0139716. doi: 10.1371/journal.pone.0139716 (PMC4592257; doi:10.1371/journal.pone.0139716)
Supplement: S1 Table — (DOCX) [file pone.0139716.s001.docx]

**Table S1.** Family-based association result for FEV_1_ in the discovery stage (participants without known respiratory disease only).

| Chr | SNP | Position* | Nearest gene | Minor Allele | MAF | *P* value |
| --- | --- | --- | --- | --- | --- | --- |
|  |  |  |  |  |  |  |
| 12 | rs12582875 | 127598647 | *TMEM132C* | A | 0.161 | 7.12 × 10^-6^ |
|  |  |  |  |  |  |  |
| 6 | rs4710230 | 167603059 | *UNC93A* | T | 0.387 | 8.49 × 10^-6^ |
|  | rs3010558 | 167616938 | *UNC93A* | T | 0.383 | 2.7 × 10^-5^ |
|  |  |  |  |  |  |  |
| 3 | rs264676 | 65861112 | *MAGI1* | G | 0.307 | 4.63 × 10^-6^ |
|  |  |  |  |  |  |  |
| 18 | rs7504607 | 73349291 | *GALR1* | C | 0.458 | 3.7 × 10^-5^ |
|  |  |  |  |  |  |  |
| 4 | rs6855113 | 121225082 | *MAD2L1* | T | 0.079 | 5.7 × 10^-6^ |
|  | rs6831851 | 121241185 | *MAD2L1* | C | 0.079 | 5.7 × 10^-6^ |

* SNP postions are based on NCBI Build 36

Chr, Chromosome; MAF, Minor allele frequency
