# Supplementary material for: Family-Based Association Study of Pulmonary Function in a Population in Northeast Asia
Source: PLoS One. 2015 Oct 2;10(10):e0139716. doi: 10.1371/journal.pone.0139716 (PMC4592257; doi:10.1371/journal.pone.0139716)
Supplement: S2 Table — (DOCX) [file pone.0139716.s002.docx]

**Table S2.** The comparison of the minor allele frequency of 7 associated SNPs in the discovery and replication study.

| Chr | SNP | Position* | Minor Allele | MAF of Discovery stage | MAF of Replication study | *P* value |
| --- | --- | --- | --- | --- | --- | --- |
|  |  |  |  |  |  |  |
| 12 | rs12582875 | 127598647 | A | 0.161 | 0.283 | < 0.001 |
|  |  |  |  |  |  |  |
| 6 | rs4710230 | 167603059 | T | 0.387 | 0.296 | < 0.001 |
|  | rs3010558 | 167616938 | T | 0.383 | 0.301 | < 0.001 |
|  |  |  |  |  |  |  |
| 3 | rs264676 | 65861112 | G | 0.307 | 0.197 | < 0.001 |
|  |  |  |  |  |  |  |
| 18 | rs7504607 | 73349291 | C | 0.458 | 0.446 | 0.402 |
|  |  |  |  |  |  |  |
| 4 | rs6855113 | 121225082 | T | 0.079 | - |  |
|  | rs6831851 | 121241185 | C | 0.079 | - |  |

* SNP postions are based on NCBI Build 36

Chr, Chromosome; MAF, Minor allele frequency
